# Synthesis of Homochiral *N*‐Heterocyclic Carbene‐Based Nanosheets for Enhanced Asymmetric Catalysis

**DOI:** 10.1002/advs.202412592

**Published:** 2024-11-19

**Authors:** Xinchao Wang, Zhiwen Wang, Zhaoxing Wang, Tian‐Fu Liu, Shangda Li, Fei Wang, Jian Zhang

**Affiliations:** ^1^ State Key Laboratory of Structural Chemistry, Fujian Institute of Research on the Structure of Matter Chinese Academy of Sciences Fuzhou Fujian 350002 P. R. China

**Keywords:** asymmetric catalysis, benzoin condensation, chiral N‐Heterocyclic carbenes, heterogeneous catalysis, metalâˆ’organic framework nanosheets

## Abstract

Utilizing ultrathin 2D metal–organic framework nanosheets (2D MONs) as supports for incorporating chiral catalysts represents a highly promising avenue in the field of asymmetric catalysis. In this study, four pairs of isostructural chiral metal–organic layers (MOLs) adorned with *N*‐Heterocyclic carbene (NHC) groups are successfully synthesized. Notably, the obtained bulky (*S*)‐1‐Zn crystals can be readily delaminated into ultrathin MONs consisting of 1–2 layers through an ion intercalation‐assisted exfoliation process. Subsequent asymmetric catalysis studies revealed that the NHC sites can be effectively activated by a proton sponge while maintaining structural integrity for the subsequent benzoin condensation. Due to their well‐exposed catalytic sites, ultrathin morphology, and porous structure, the (*S*)‐1‐Zn nanosheets exhibited significantly enhanced yield and enantioselectivity compared to their bulk counterparts and organic precursors. This research highlights an efficient strategy for incorporating chiral NHC species onto 2D MONs, thereby unlocking their immense potential for heterogeneous asymmetric catalysis.

## Graphical abstract

2D nanomaterials present promising platforms for supporting chiral catalysts. This paper explores an example of ultrathin metal–organic nanostructures (MONs) constructed with chiral N‐heterocyclic carbenes (NHCs) as spacers, which can significantly enhance enantioselectivity in asymmetric benzoin condensation reactions.

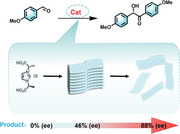

## 1 Introduction

Asymmetric catalysis has emerged as a fundamental approach for synthesizing enantiomerically pure chiral compounds, which holds immense value for both scientific research and industrial applications.^[^ ^1^ ^]^ Traditionally, most asymmetric catalytic reactions in practical settings have been conducted in homogeneous systems, which suffer from drawbacks such as high costs, difficulties in recycling, catalyst deactivation, and complicated separation processes.^[^ ^2^ ^]^ To address these challenges, immobilizing homogeneous catalysts onto porous substrates has emerged as a highly promising solution.^[^ ^3^ ^]^ Generally, an ideal support should exhibit strong interactions with catalytic species to prevent leaching while providing well‐exposed and easily accessible catalytic sites for reactants. In recent years, 2D nanomaterials have garnered significant research attention and undergone rapid development.^[^ ^4^ ^]^ Their high external surface areas, ultrathin morphology, and high affinity for reactant substrates and catalytic species enable the exposure of numerous active sites and facilitate fast mass transport, leading to enhanced catalytic efficiency.^[^ ^5^ ^]^ From this perspective, 2D nanomaterials hold great promise as catalyst supports for heterogeneous asymmetric catalysis. Among them, metal–organic framework nanosheets (MONs), a novel category of 2D nanomaterials, achieved by reducing the dimensionality of 3D metal–organic frameworks (MOFs) or metal–organic layers (MOLs) to a single or few layers with a sheet‐like morphology.^[^ ^6^ ^]^ MONs not only retain the benefits of 2D nanomaterials but also integrate the advantages of MOFs, such as high porosity, large internal surface area, and modular structures. The combination of these features enables the rational design of catalysts to meet various demands, and some have already demonstrated enhanced catalytic performance compared to their 3D counterparts.^[^ ^7^ ^]^ Specifically, if intrinsic chiral building blocks can be embedded in MONs, these compounds may outperform their corresponding 3D counterparts in asymmetric catalysis.^[^ ^8^ ^]^ For instance, Cui and colleagues reported pioneering progress in creating ultrathin chiral MONs for catalyzing asymmetric condensation and amine addition in 2019.^[^ ^8b^ ^]^ They discovered that enhanced enantioselectivity can be achieved by integrating chiral binol/biphenol into MONs compared to their bulky 3D counterparts or organic precursors. Thus, the impressive catalytic performance and unique chiral compatibility of MONs underscore their immense potential in heterogeneous asymmetric catalysis.

**Scheme 1:**
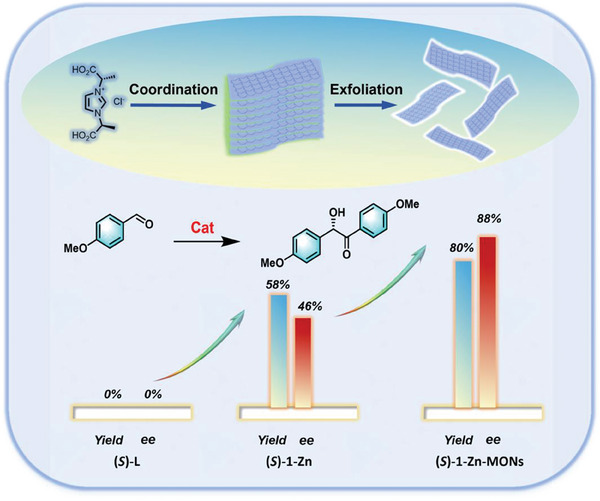
The schematic diagram illustrates the catalytic results (bottom).

Inspired by aforementioned background, we are motivated to utilize MONs as platforms for incorporating chiral ligands *N*‐heterocyclic carbenes (NHCs), which usually suffer from side reactions and deactivation issues (caused by dimerization) during homogeneous catalytic reactions.^[^ ^9^ ^]^ In this study, the homochiral ligand 1,3‐bis((*S*)‐1‐carboxyethyl)‐1H‐imidazol‐3‐ium chloride (*S*)‐L and its enantiomer (*R*)‐L were respectively self‐assembled with various metal ions, including Zn^2^⁺, Co^2^⁺, Mn^2^⁺, and Cd^2^⁺ through solvothermal reactions. This approach successfully yielded four pairs of isomorphic chiral MOLs, featuring uniformly distributed NHCs on skeletons (**Scheme** **1**). Furthermore, the bulky crystals of (*S*)‐1‐Zn can be delaminated into ultrathin (1‐2 layers) nanosheets using ion intercalation‐assisted ultrasonic exfoliation technique. Subsequent catalysis studies showed that, compared to their bulky counterparts or organic precursors, the ultrathin nanosheets of (*S*)‐1‐Zn exhibited superior catalytic efficiency with enhanced yield and enantioselectivity for benzoin condensation reaction. Notably, the reaction can be successfully scaled up to the gram level without diminishing its catalytic performance. This study introduces an innovative approach for incorporating chiral NHCs precursors within porous 2D nanomaterials, which demonstrates their potential for practical applications in heterogeneous asymmetric catalysis.

## 2 Results and Discussion

**Figure 1:**
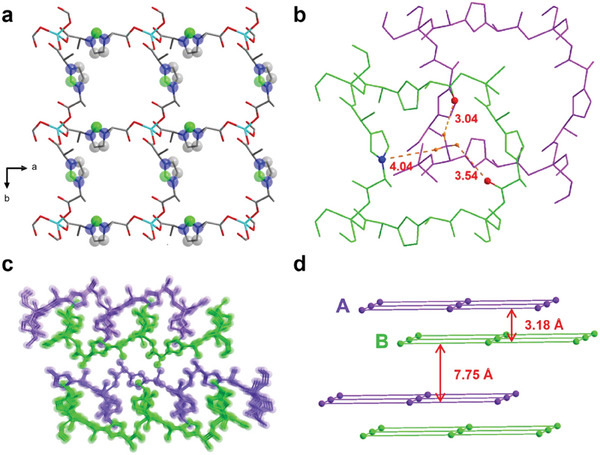
a) Layer structure of (*S*)‐1‐Zn. turquoise, Zn; blue, N; gray, C; green, C active site; red, O; b) Feeble hydrogen bonding between adjacent layers; dashed lines indicate O─H or N─H internuclear distances from 3.04 to 4.04 Å; c) The stack mode between adjacent layers of (*S*)‐1‐Zn. d) The simplified stack mode and the distance between adjacent layers.

The homochiral imidazolium‐based dicarboxylate ligand, specifically 1,3‐bis((*S*)‐1‐carboxyethyl)‐1H‐imidazol‐3‐ium chloride and its enantiomer, designated as (*S*)‐L and (*R*)‐L, were efficiently synthesized from optically pure alanine via the Debus‐Radziszewski reaction (Figure S1, Supporting Information). Subsequently, solvothermal reactions involving either (*S*)‐L or (*R*)‐L and the corresponding metal salts were conducted within a temperature range of 80–120 °C in solvents such as DMF or DMSO. Comprehensive reaction conditions for crystal growth are detailed in the Supporting Information. Ultimately, four pairs of compounds were successfully isolated (**Table** **1**). Their structural configurations were precisely elucidated through Single‐crystal X‐ray diffraction (SXRD). Data analysis revealed that all eight compounds exhibit isostructural features, belonging to the orthorhombic crystal system with the chiral space group P2_1_2_1_2_1_ (Tables S1 and S2, Supporting Information). To avoid repetition, (*S*)‐1‐Zn is selected as a representative to elucidate their common structural features. Within the framework of (*S*)‐1‐Zn, the metal centers are tetrahedrally coordinated by O atoms derived from four individual ligands, adhering to a monodentate *κ* ^1^ coordination mode (Figure S2, Supporting Information). The charge neutrality is maintained without the necessity for additional counter anions, as the metal ions balance the ligand charges. Furthermore, each (*S*)‐L ligand serves as a μ_2_ bridge, linking two metal centers through its bilateral carboxy groups, thereby constructing an extensive 2D layer structure (**Figure** **1**a). Additionally, each metal unit can be conceptualized as a 4‐connected node, simplifying the overall framework into a 4‐connected topological network (Figures S3 and S4, Supporting Information). Upon further organization, these crystal units stack into layered architectures, with the metal centers facing the interlayer spaces. The hydrogen atoms located on the methyl group within the framework engage in the formation of feeble hydrogen bonds with N and O atoms present in adjacent layers, with bond lengths ranging from 3.04 to 4.04 Å (Figure 1b). Furthermore, the stacking arrangement between these layers is achieved in an AB‐type configuration (Figure 1c). Notably, layers A and B interpenetrate, maintaining a spacing of 3.18 Å, while the distance between adjacent AB layers ≈7.75 Å (Figure 1d). Besides, the spectra of conducted circular dichroism (CD) tests with each pair of crystals showed mirror images, confirming the chiral characteristics of these compounds (Figure S5, Supporting Information).

**Table 1:** Crystal data.

Compounds	Empirical formula	Space group	a/Å	b/Å	c/Å	Volume [Å^3^]	Flack parameter	CCDC number
(*S*)‐1‐Zn	C_18_H_22_N_4_O_8_Zn	*P*2_1_2_1_2_1_	9.94250(10)	10.0165(2	21.8950(4)	2180.50(6)	−0.023(14)	2 362 667
(*R*)‐1‐Zn	C_18_H_22_N_4_O_8_Zn	*P*2_1_2_1_2_1_	9.9437(2)	10.0181(3)	21.9042(9)	2182.03(12)	−0.008(4)	2 362 833
(*S*)‐1‐Co	C_18_H_22_N_4_O_8_Co	*P*2_1_2_1_2_1_	9.8613(3)	9.9033(3)	21.6822(10)	2117.47(13)	0.008(9)	2 367 558
(*R*)‐1‐Co	C_18_H_22_N_4_O_8_Co	*P*2_1_2_1_2_1_	9.8520(4)	9.9104(4)	21.6896(14)	2117.71(18)	0.012(10)	2 367 559
(*S*)‐1‐Mn	C_18_H_22_N_4_O_8_Mn	*P*2_1_2_1_2_1_	9.7743(2)	9.7743(2)	20.7890(3)	2024.46(5)	−0.002(3)	2 367 556
(*R*)‐1‐Mn	C_18_H_22_N_4_O_8_Mn	*P*2_1_2_1_2_1_	9.7788(2)	9.9695(2)	20.8572(5)	2033.36(8)	−0.013(6)	2 367 557
(*S*)‐1‐Cd	C_18_H_22_N_4_O_8_Cd	*P*2_1_2_1_2_1_	10.0359(2)	20.8638(4)	9.8304(2)	2058.36(7)	−0.032(10)	2 367 560
(*R*)‐1‐Cd	C_18_H_22_N_4_O_8_Cd	*P*2_1_2_1_2_1_	9.8435(2)	10.0428(2)	20.8644(5)	2062.58(8)	−0.029(11)	2 362 672

**Figure 2:**
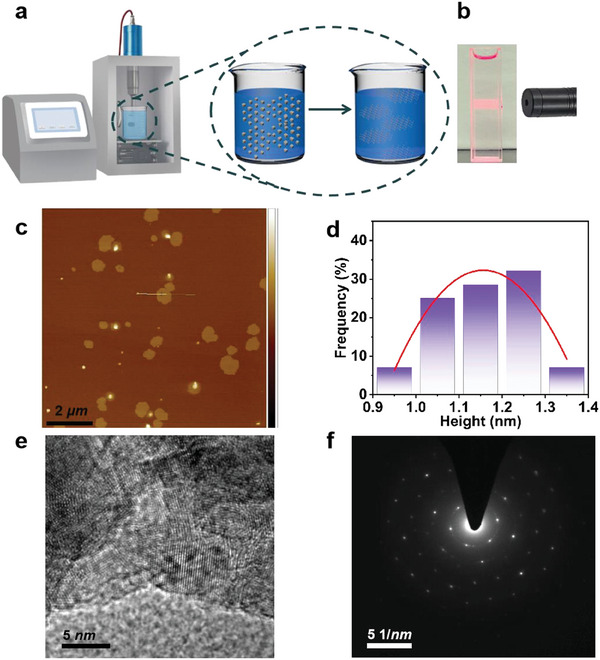
Crystal microstructure of (*S*)‐1‐Zn nanosheets. a) Stripping diagram. b) The Tyndall effect of (*S*)‐1‐Zn nanosheets colloidal suspension. c) Atomic force microscopy (AFM) image, and thickness of AFM image for the selective area. d) Atomic force microscopy (AFM) image for the selective area. e) High resolution transmission electron microscope (HRTEM) image. f) Selected area electron diffraction (SAED) pattern.

Notably, these frameworks exhibit remarkable structural robustness, enduring both solvent and thermal challenges. For instance, the integrity of the (*S*)‐1‐Zn framework remains intact even after a seven‐day immersion in diverse common organic solvents, including DMF, EtOH, and DMSO, as corroborated by PXRD analysis (Figure S6, Supporting Information). Thermal stability assessments via Thermal Gravimetric Analysis (TGA) reveal that (*S*)‐1‐Zn crystals, after thorough washing with DCM, preserve their structural integrity up to 300 °C (Figure S11, Supporting Information). Furthermore, these compounds demonstrate outstanding long‐term stability, with PXRD spectra of (*S*)‐1‐Zn remaining consistent after storage in DMF, EtOH, or exposure to air for over 3 months (Figure S7, Supporting Information). Complementing these findings, Fourier transform infrared spectroscopy (FTIR) analysis of (*S*)‐1‐Zn crystals immersed in EtOH for 3 months confirms the unwavering presence of key characteristic peaks, including those associated with pre‐NHCs sites (at 3145 cm^−1^), indicating no structural alterations (Figure S12, Supporting Information). Notably, the other three compounds exhibit similar but slightly inferior stability profiles to (*S*)‐1‐Zn, with detailed information provided in the Supporting Information (Figures S8–S10, Supporting Information). The robustness of the compounds presents ample opportunities for morphology modulation, aiming to alleviate diffusion limitations and enhance catalytic activity. To achieve this, we strategically transitioned from manipulating bulky solids to fabricating ultrathin 2D nanosheets. Upon analyzing the structure of (*S*)‐1‐Zn, it is postulated that the existence of feeble interlayer forces between adjacent layers renders it amenable to delamination via mechanical force. Delightfully, adopting the ion intercalation‐assisted liquid‐phase exfoliation method (schematic depicted in **Figure** **2**a), we successfully transformed the bulky (*S*)‐1‐Zn crystals into uniform ultrathin nanosheets, which were subsequently subjected to rigorous characterization. Initially, the suspension under laser irradiation exhibited pronounced Tyndall scattering (Figure 2b), indicative of the excellent colloidal dispersion of (*S*)‐1‐Zn nanosheets. Then the microscopic morphology of the (*S*)‐1‐Zn‐MONs was illustrated by electron microscopy. With the scanning electron microscope (SEM) image, wrinkled free‐standing nanosheets of 2.4 × 3.2 µm^2^ were detected (Figure S13, Supporting Information). The atomic force microscopy (AFM) analysis corroborated the ultrathin nature of these delaminated nanosheets, with a thickness of ≈1.2 nm (equivalent to 1–2 layers) (Figure 2c,d). Moreover, the distinct lattice stripes and single‐crystal diffraction patterns validated the outstanding single‐crystal structure of (*S*)‐1‐Zn‐MONs (Figure 2e). Notably, the selected area electron diffraction (SAED) pattern affirmed that the (*S*)‐1‐Zn‐MONs retained their pristine crystal form, exhibiting a characteristic orthorhombic single‐crystal structure (Figure 2f).

Encouraged by the stability and processability of these pre‐NHC‐decorated crystals, we embarked on exploring their catalytic prowess in asymmetric benzoin condensation reactions. This pathway efficiently furnishes optically pure α‐hydroxyketones (acyloins), which are invaluable intermediates across pharmaceutical, agrochemical, and materials science industries.^[^ ^10^ ^]^ Employing *p*‐anisaldehyde as the starting material, we initiated our investigation with the unexfoliated bulky solid of (*S*)‐1‐Zn in EtOH at 60 °C under a N_2_ atmosphere. Initially, focusing on activating the NHC catalytic sites from imidazolium salts via deprotonation, we meticulously selected suitable bases, mindful of the potential for framework decomposition in strongly alkaline solutions. Our experiments revealed that common inorganic or organic bases like K_2_CO_3_, KOH, Et_3_N, or *^i^* Pr_2_NEt were ineffective for this transformation (Table S3, Supporting Information). However, the adoption of DBU successfully promoted the reaction, yielding the desired product **1a** in 28% yield (**Table** **2**, entry 1). Further screenings identified proton sponge as an optimal base due to its exceptionally suitable alkalinity properties, enhancing the yield to 47% with 45% enantiomeric excess (ee) (entry 2). To further bolster catalytic efficiency, we introduced 1‐ethyl‐3‐methylimidazolium chloride as a cocatalyst, which improved the yield to 58% while maintaining comparable enantioselective control (entry 3). Under similar conditions, three other crystals were compared with (*S*)‐1‐Zn, but their catalytic performances fell short (entries 4–6). We hypothesize that the superior stability exhibited by (S)‐1‐Zn, in comparison to the other three crystalline materials, serves as a critical factor underlying its preeminence as a catalyst candidate. Notably, control experiments with the free ligand (*S*)‐1 yielded no product, even with the addition of Zn(NO_3_)_2_·6H_2_O (entries 7–8), underscoring the crucial role of the catalyst's framework crystallinity. Remarkably, when nanosheets of (*S*)‐1‐Zn, exfoliated for 1 h, were employed, a significant leap in catalytic performance was achieved, affording the desired **1a** in 74% yield with 67% ee (entry 9). This underscores the advantage of the ultrathin nanosheet structure in enhancing catalytic activity and selectivity. Furthermore, by extending the exfoliation time to 2 h, the resulting (*S*)‐1‐Zn‐MONs demonstrated their utmost catalytic efficiency, boosting the yield to 82% while achieving an optimal enantiomeric excess of 88% (entry 10). Notably, further prolonging the delamination period to 3 h did not yield superior results (entry 11), thus confirming the 2 h exfoliated (*S*)‐1‐Zn‐MONs as the optimal catalyst. Subsequently, we reassessed the influence of additives and discovered that the absence of 1‐ethyl‐3‐methylimidazolium chloride led to a moderate decrease in yield, while the ee value remained largely unaffected (entry 12). Operating under these optimized reaction conditions, we successfully scaled up the reaction to 8 mmol, smoothly yielding 1.63 g of chiral product **1a** in 75% yield and 86% ee (entry 13). Subsequently, upon completion of the experiment, the heterogeneous catalyst was effortlessly separated from the reaction mixture through simple centrifugation. Powder X‐ray diffraction (PXRD) analysis verified that the recovered catalyst retained its pristine crystalline state (Figure S14, Supporting Information). Notably, the recycled catalyst remained effective for three consecutive cycles, with the results summarized in Table 2 (bottom‐a). Lastly, we expanded our investigation to include other substrates with diverse substituents on the aromatic ring (Table 2, bottom‐b). Encouragingly, using *p*‐tolualdehyde as the starting material, we achieved an impressive ee of up to 91% for product **1c**, demonstrating the versatility and high enantioselectivity of our catalytic system.

**Table 2:** Condition optimizations for asymmetric benzoin condensation.^a)^

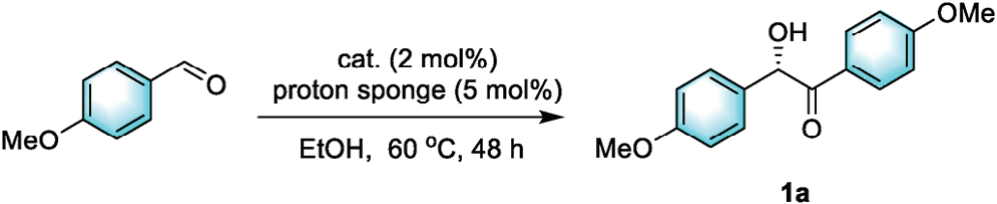			
Entry	Cat.	Yield [%]	ee [%]
1^b)^	Bulky (*S*)‐1‐Zn	28	11
2	Bulky (*S*)‐1‐Zn	47	45
3^c)^	Bulky (*S*)‐1‐Zn	58	46
4^c)^	Bulky (*S*)‐1‐Mn	40	3
5^c)^	Bulky (*S*)‐1‐Co	42	20
6^c)^	Bulky (S)‐1‐Cd	50	40
7	(*S*)‐L	N.D.	‐
8	(*S*)‐1 + Zn(NO_3_)_2_·6H_2_O	N.D.	‐
9^c)^, ^d)^	(*S*)‐1‐Zn‐MONs	74	67
10^c)^, ^e)^	(*S*)‐1‐Zn‐MONs	82	88
11^c)^, ^f)^	(*S*)‐1‐Zn‐MONs	80	88
12^e)^	(*S*)‐1‐Zn‐MONs	70	86
13^c)^, ^e)^, ^g)^	(*S*)‐1‐Zn‐MONs	75	86
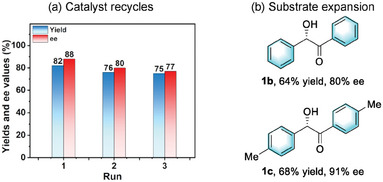			

^a)^ Reaction conditions: *p*‐Anisaldehyde (2 mmol, 1.0 equiv), cat. (0.04 mmol, 2 mol%), proton sponge (0.1 mmol, 5 mol%), EtOH (2 mL), N_2_ atmosphere, 60 °C, 48 h. Isolated yields was reported. Enantiomeric ratios of **1a** were determined by HPLC analysis on a chiral stationary phase;

^b)^ DBU as the base;

^c)^ 1‐Ethyl‐3‐methylimidazolium chloride (1 mmol%) was add an additive;

^d)^ Exfoliation for 1 h;

^e)^ Exfoliation for 2 h;

^f)^ Exfoliation for 3 h;

^g)^ Reaction scale: 8 mmol. Proton sponge: 1,8‐bis(dimethylamino)naphthalene. N.D.: not detected.

**Figure 3:**
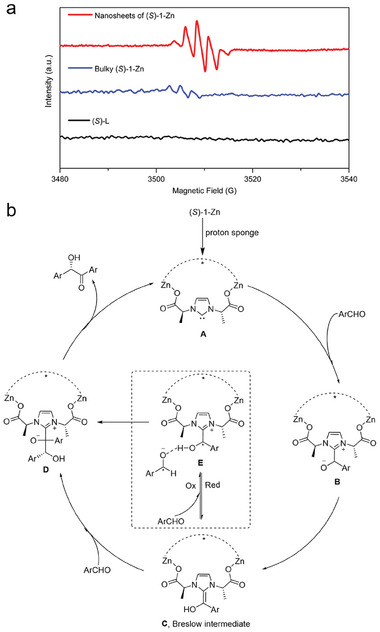
a) In situ EPR tests of the reaction systems catalyzed by (*S*)‐1‐Zn‐MONs, bulky (*S*)‐1‐Zn and (*S*)‐L. b) Tentative catalytic cycle for (*S*)‐1‐Zn catalyzed asymmetric benzoin condensation.

To gain insights into the disparity in catalytic activities between ultrathin 2D MONs and their bulk counterparts, as well as their organic precursors, we conducted a series of mechanistic experiments. Given that radical transients have been reported to coexist with Breslow intermediates during NHC‐catalyzed benzoin condensation,^[^ ^11^ ^]^ we devised a strategy to confirm the in situ generation of radical species using electron paramagnetic resonance (EPR) spectroscopy. Our EPR inspection findings are presented in **Figure** **3**a. Notably, a silent EPR spectrum was observed when only the organic precursor (*S*)‐1 was employed as the catalyst (Figure 3a, black). In contrast, moderate EPR peaks emerged when the bulk (*S*)‐1‐Zn was used (blue). Remarkably, the EPR signals intensified significantly when nanosheets of (*S*)‐1‐Zn were utilized (red). These EPR signals were attributed to the transient radical pair generated during the catalytic cycles, and their intensities correlated well with the observed catalytic outcomes. Based on the EPR analysis and previous research findings, we propose a tentative catalytic cycle for the (*S*)‐1‐Zn‐catalyzed asymmetric benzoin condensation in Figure 3b. Initially, the carbene sites of (*S*)‐1‐Zn are exposed through deprotonation with a proton sponge, yielding an active catalyst species **A**. Subsequently, **A** engages in an electrophilic attack with the corresponding aromatic aldehyde to form the primary adduct **B**, which then undergoes hydrogen atom transfer to generate the Breslow intermediate **C**. The transformation of **C** to **D** involves non‐unique pathways, with one hypothesis suggesting the involvement of a radical pair **E** in the benzoin formation process, as supported by our in situ EPR analysis. Finally, cleavage of the C‐C bond in **D** produces chiral α‐hydroxyketones and regenerates the active species **A**. It is important to note that the reversibility of one or more steps in the proposed catalytic cycle remains unclear, and alternative catalytic mechanisms cannot be completely ruled out at this stage.

## 3 Conclusion

An exemplary chiral NHC‐based molecular catalyst was successfully anchored within MOLs via solvothermal coordination polymerization. The resulting four pairs of isostructural chiral crystalline layers exhibited robust stability across thermal and solvent challenges. Notably, the bulky (*S*)‐1‐Zn crystals were skillfully delaminated into ultrathin nanosheets through an ion intercalation‐facilitated liquid‐phase ultrasonic exfoliation process. Although the organic precursor (*S*)‐1 was ineffective in catalyzing asymmetric benzoin condensation, the integrated frameworks, aided by a proton sponge, efficiently promoted the reaction. Critically, the ultrathin (*S*)‐1‐Zn nanosheets significantly enhanced enantioselectivity, surpassing from 46% to 88%. In situ EPR analysis illuminated the distinct catalytic activities, prompting the formulation of a tentative catalytic cycle. This study pioneers a fresh approach to immobilizing chiral NHC species within 2D nanomaterials. Our laboratory remains committed to broadening the application realm of chiral MONs in heterogeneous asymmetric catalysis.

## Conflict of Interest

The authors declare no conflict of interest

## Supporting information



Supporting Information

Supporting Information

## Data Availability

The data that support the findings of this study are available in the supplementary material of this article.
