# Supplementary material for: Synthesis of Homochiral N‐Heterocyclic Carbene‐Based Nanosheets for Enhanced Asymmetric Catalysis
Source: Adv Sci (Weinh). 2024 Nov 19;12(2):2412592. doi: 10.1002/advs.202412592 (PMC11727139; doi:10.1002/advs.202412592)
Supplement: Supplementary file 1 — Supporting Information [file ADVS-12-2412592-s001.docx]

**Supporting Information**

Homochiral NHC-based Metal−Organic Framework Nanosheets for Asymmetric Catalysis

Xinchao Wang^[a]^, Zhiwen Wang^[a]^, Zhaoxing Wang^[a]^, Tian-Fu Liu^[a]^, Shangda Li*^[a]^, Fei Wang*^[a]^, and Jian Zhang*^[a]^

[a] State Key Laboratory of Structural Chemistry, Fujian Institute of Research on the Structure of Matter, Chinese Academy of Sciences Fuzhou, Fujian 350002, P. R. China

E-mail: [sdli@fjirsm.ac.cn](mailto:sdli@fjirsm.ac.cn), [wangfei04@fjirsm.ac.cn](mailto:wangfei04@fjirsm.ac.cn), zhj@fjirsm.ac.cn.

**Table of Contents**

[Supporting Information 1](#_Toc10984)

[1. Materials and Methods 2](#_Toc32044)

[2. Synthesis of Ligand (*S*)-L 2](#_Toc21067)

[3. Synthesis of Compounds 4](#_Toc1946)

[4. Structure analysis and basic characterization 5](#_Toc16467)

[5. Exfoliation of (*S*)-1-Zn 14](#_Toc26662)

[6. Asymmetric Catalysis 15](#_Toc9958)

[7. EPR test 25](#_Toc877)

[8. Reference 26](#_Toc12594)

# 1. Materials and Methods

The reagents and solvents utilized in this work are commercially accessible and do not necessitate additional purification. Fourier Transform Infrared (FT-IR) spectroscopies information in the range of 500-4000 cm^-1^ was recorded on an ABB Bomem MB102 spectrometer. The Circular Dichroism (CD) spectroscopy data was collected on an MOS-450 spectropolarimeter. Thermogravimetric analysis (TGA) was conducted on a STA449C integrated thermal analyzer, with a heating rate of 10 °C/min in a nitrogen atmosphere. The collection of Powder X-ray diffraction (PXRD) data was performed using a Rigaku Mini Flex II diffractometer with Cu-K*α* radiation. The enantiomeric excess (ee) values in the catalytic reactions were quantified using a high-performance liquid chromatography (HPLC) system, specifically the Agilent 1200 LC equipment. The CHIRALCEL OD-H analytical column (4.6 mm × 250 mm) was provided by Daicel Corporation. The exfoliation experiments were performed on a Branson Sonifler 250D instrument, maximum power: 250 W, standard frequency: 20 KHz. Scanning electron microscope (SEM) images taken by ZEISS Gemini 300 are used to observe the microscopic morphology of the crystals. Transmission Electron Microscopy (TEM) images were acquired using a Talos F200X instrument, while the Atomic Force Microscopy (AFM) images were obtained using a Dimension ICON, and both TEM and AFM samples were ultrasonically dispersed in ethanol at a ratio of 1 mg/mL for 15 minutes. Crystallographic data were collected on a Rigaku Synergy Custom (Liquid MetalJet D2+) diffractometer with Ga K*α* radiation (λ = 1.3405 Å). The structures were solved with direct methods using OLEX2 and refined by full-matrix least-squares on F^2^ using SHELXTL.

# 2. Synthesis of Ligand (*S*)-L

Under air atmosphere and room temperature, L-alanine (5 mmol, 445 mg) and paraformaldehyde (5 mmol, 150 mg) were added to toluene (20 mL). After stirring at room temperature for 30 min, the mixture was cooled to 0°C, followed by the addition of another 5 mmol (445 mg) of L-alanine. After stirring for 15 minutes, 37% HCl (5 mmol, 0.42 mL) was slowly added to the mixture. The reaction mixture was then allowed to warm gradually to room temperature, followed by the addition of Glyoxal (40% in H_2_O) (5 mmol, 0.725 mL). The resulting solution was stirred for an additional 30 minutes. Subsequently, the mixture was heated to 60 °C and stirred overnight to complete the reaction. Once the reaction was deemed complete, the reaction mixture was cooled back to room temperature and concentrated under reduced pressure to obtain the crude compound. To purify the product, a small amount of ether and an appropriate volume of ethanol were added and mixed thoroughly. Through recrystallization, (*S*)-1 was isolated as a white solid in 82% yield (1.02 g). ^1^H NMR (400 MHz, D_2_O) δ 8.87 (s, 1H), 7.46 (s, 2H), 5.06 (q, *J* = 7.4 Hz, 2H), 1.69 (d, *J* = 7.4 Hz, 6H); ^13^C NMR (101 MHz, D_2_O) δ 173.70 (d, *J* = 3.6 Hz), 135.37, 121.60, 58.98, 17.11.

The enantiomer (*R*)-L was synthesized following the same operation sequences with D-alanine as the starting material.

**Figure S1**. ^1^H and ^13^C NMR spectra of (*S*)-L.

# 3. Synthesis of Compounds

**(*S*)-1-Zn:** A mixture of (*S*)-L (49.6 mg, 0.2 mmol), Zn(NO_3_)_2_·6H_2_O (94 mg, 0.4 mmol) and DMF (3 mL) was sealed in a 20 mL vial and transferred to a preheated oven at 100 ^o^C for 2 days. When cooled to room temperature, colorless block crystals were obtained (yield: 64% based on (*S*)-L). The crystals are rinsed with DMF and preserved under a sealed and dry environment.

**(*S*)-1-Zn-Co:** A mixture of (*S*)-L (49.6 mg, 0.2 mmol), CoCl_2_ (52 mg, 0.4 mmol) and DMF (3 mL) was sealed in a 20 mL vial and transferred to a preheated oven at 100 ^o^C for 2 days. When cooled to room temperature, colorless block crystals were obtained (yield: 46% based on (*S*)-L). The crystals are rinsed with DMF and preserved under a sealed and dry environment.

**(*S*)-1-Mn:** A mixture of (*S*)-1 (49.6 mg, 0.2 mmol), Mn(OAc)_2_·4H_2_O (98 mg, 0.4 mmol) and DMSO (3 mL) was sealed in a 20 mL vial and transferred to a preheated oven at 120 ^o^C for 2 days. When cooled to room temperature, colorless block crystals were obtained (yield: 52% based on (*S*)-L). The crystals are rinsed with DMSO and preserved under a sealed and dry environment.

**(*S*)-1-Cd:** A mixture of (*S*)-1 (49.6 mg, 0.2 mmol), Cd(NO_3_)_2_·4H_2_O (123 mg, 0.4 mmol) and DMF (3 mL) was sealed in a 20 mL vial and transferred to a preheated oven at 100 ^o^C for 2 days. When cooled to room temperature, colorless block crystals were obtained (yield: 31% based on (*S*)-L). The crystals are rinsed with DMF and preserved under a sealed and dry environment.

# 4. Structure analysis and basic characterization

## 4.1 Crystal data and structure refinement

**Table S1.** Crystal data and structure refinement

| Identification code | (*S*)-1-Zn | (*S*)-1-Co | (*S*)-1-Mn | (*S*)-1-Cd |
| --- | --- | --- | --- | --- |
| Empirical formula | C_18_H_22_N_4_O_8_Zn | C_18_H_22_CoN_4_O_8_ | C_18_H_22_MnN_4_O_8_ | C_18_H_22_CdN_4_O_8_ |
| Formula weight | 487.76 | 481.32 | 477.33 | 534.79 |
| Temperature/K | 179(7) | 100.00(10) | 100.01(16) | 100.15 |
| Crystal system | orthorhombic | orthorhombic | orthorhombic | orthorhombic |
| Space group | *P*2_1_2_1_2_1_ | *P*2_1_2_1_2_1_ | P2_1_2_1_2_1_ | P2_1_2_1_2_1_ |
| a/Å | 9.94250(10) | 9.8613(3) | 9.7743(2) | 10.0359(2) |
| b/Å | 10.0165(2) | 9.9033(3) | 9.96300(10) | 20.8638(4) |
| c/Å | 21.8950(4) | 21.6822(10) | 20.7890(3) | 9.8304(2) |
| α/° | 90 | 90 | 90 | 90 |
| β/° | 90 | 90 | 90 | 90 |
| γ/° | 90 | 90 | 90 | 90 |
| Volume/Å^3^ | 2180.50(6) | 2117.47(13) | 2024.46(5) | 2058.36(7) |
| Z | 4 | 4 | 4 | 4 |
| ρ_calc_g/cm^3^ | 1.486 | 1.510 | 1.566 | 1.726 |
| μ/mm^‑1^ | 1.324 | 0.863 | 3.958 | 1.115 |
| F(000) | 1008.0 | 996.0 | 988.0 | 1080.0 |
| Index ranges | -12 ≤ h ≤ 12, -12 ≤ k ≤ 12, -28 ≤ l ≤ 28 | -11 ≤ h ≤ 11, -11 ≤ k ≤ 11, -26 ≤ l ≤ 26 | -11 ≤ h ≤ 8, -12 ≤ k ≤ 11, -25 ≤ l ≤ 25 | -14 ≤ h ≤ 14, -29 ≤ k ≤ 28, -13 ≤ l ≤ 13 |
| Reflections collected | 26966 | 24403 | 19944 | 31574 |
| Independent reflections | 4916 [R_int_ = 0.0548, R_sigma_ = 0.0343] | 3921 [R_int_ = 0.0434, R_sigma_ = 0.0277] | 3995 [R_int_ = 0.0421, R_sigma_ = 0.0293] | 5561 [R_int_ = 0.0396, R_sigma_ = 0.0297] |
| Data/restraints/parameters | 4916/198/284 | 3921/86/313 | 3995/0/284 | 5561/0/284 |
| Goodness-of-fit on F^2^ | 1.089 | 1.029 | 1.045 | 1.085 |
| Final R indexes [I>=2σ (I)] | R_1_ = 0.0512, wR_2_ = 0.1539 | R_1_ = 0.0855, wR_2_ = 0.2430 | R_1_ = 0.0262, wR_2_ = 0.0654 | R_1_ = 0.0241, wR_2_ = 0.0489 |
| Final R indexes [all data] | R_1_ = 0.0581, wR_2_ = 0.1609 | R_1_ = 0.1024, wR_2_ = 0.2610 | R_1_ = 0.0287, wR_2_ = 0.0666 | R_1_ = 0.0285, wR_2_ = 0.0500 |
| Flack parameter | -0.023(14) | 0.008(9) | -0.002(3) | -0.032(10) |
| CCDC number | 2362667 | 2367558 | 2367556 | 2367560 |

| Identification code | (*R*)-1-Zn | (*R*)-1-Co | (*R*)-1-Mn | (*R*)-1-Cd |
| --- | --- | --- | --- | --- |
| Empirical formula | C_18_H_22_N_4_O_8_Zn | C_18_H_22_CoN_4_O_8_ | C_18_H_22_MnN_4_O_8_ | C_18_H_22_CdN_4_O_8_ |
| Formula weight | 487.76 | 481.32 | 477.33 | 534.79 |
| Temperature/K | 297.40(10) | 100.00(10) | 100.00(10) | 100 |
| Crystal system | orthorhombic | orthorhombic | orthorhombic | orthorhombic |
| Space group | *P*2_1_2_1_2_1_ | *P*2_1_2_1_2_1_ | P2_1_2_1_2_1_ | P2_1_2_1_2_1_ |
| a/Å | 9.9437(2) | 9.8520(4) | 9.7788(2) | 9.8435(2) |
| b/Å | 10.0181(3) | 9.9104(4) | 9.9695(2) | 10.0428(2) |
| c/Å | 21.9042(9) | 21.6896(4) | 20.8572(5) | 20.8644(5) |
| α/° | 90 | 90 | 90 | 90 |
| β/° | 90 | 90 | 90 | 90 |
| γ/° | 90 | 90 | 90 | 90 |
| Volume/Å^3^ | 2180.50(6) | 2117.71(18) | 2033.36(8) | 2062.58(8) |
| Z | 4 | 4 | 4 | 4 |
| ρ_calc_g/cm^3^ | 1.485 | 1.510 | 1.559 | 1.722 |
| μ/mm^‑1^ | 1.177 | 0.863 | 0.704 | 1.113 |
| F(000) | 1008.0 | 996.0 | 988.0 | 1080.0 |
| Index ranges | -12 ≤ h ≤ 13, -14 ≤ k ≤ 14, -29 ≤ l ≤ 29 | -11 ≤ h ≤ 11, -11 ≤ k ≤ 11, -25 ≤ l ≤ 25 | -13 ≤ h ≤ 13,  -12 ≤ k ≤ 14,  -29 ≤ l ≤ 27 | -12 ≤ h ≤ 13,  -12 ≤ k ≤ 10,  -27 ≤ l ≤ 29 |
| Reflections collected | 31386 | 23192 | 27014 | 27263 |
| Independent reflections | 5830 [R_int_ = 0.0305, R_sigma_ = 0.0251] | 3730 [R_int_ = 0.0506, R_sigma_ = 0.0312] | 5409 [R_int_ = 0.0391, R_sigma_ = 0.0338] | 5429 [R_int_ = 0.0366, R_sigma_ = 0.0291] |
| Data/restraints/parameters | 5830/37/313 | 3730/376/324 | 5409/0/284 | 5429/0/284 |
| Goodness-of-fit on F^2^ | 1.028 | 1.045 | 1.061 | 1.056 |
| Final R indexes [I>=2σ (I)] | R_1_ = 0.0414, wR_2_ = 0.1135 | R_1_ = 0.0917, wR_2_ = 0.2519 | R_1_ = 0.0262, wR_2_ = 0.0654 | R_1_ = 0.0243, wR_2_ = 0.0507 |
| Final R indexes [all data] | R_1_ = 0.0634, wR_2_ = 0.1230 | R_1_ = 0.1104, wR_2_ = 0.2704 | R_1_ = 0.0288, wR_2_ = 0.0624 | R_1_ = 0.0282, wR_2_ = 0.0519 |
| Flack parameter | -0.008(4) | 0.0012(10) | -0.013(6) | -0.029(11) |
| CCDC number | 2362833 | 2367559 | 2367557 | 2362672 |

**Table S2.** Selected bond lengths

| (*S*)-1-Zn | | | (*S*)-1-Co | | |
| --- | --- | --- | --- | --- | --- |
| Atom | Atom | Length/Å | Atom | Atom | Length/Å |
| Zn1 | O3 | 1.953(3) | Co | O3 | 2.010(10) |
| Zn1 | O2 | 1.980(4) | Co | O4 | 1.980(7) |
| Zn1 | O7 | 1.938(4) | Co | O7 | 1.900(2) |
| Zn1 | O5 | 1.913(5) | Co | O8 | 1.988(17) |
| (*S*)-1-Cd | | | (*S*)-1-Mn | | |
| Atom | Atom | Length/Å | Atom | Atom | Length/Å |
| Cd16 | O31 | 2.445(2) | Mn | O1 | 2.2773(19) |
| Cd16 | O5 | 2.404(2) | Mn | O2 | 2.1236(19) |
| Cd16 | O19 | 2.189(2) | Mn | O4 | 2.311(2) |
| Cd16 | O30 | 2.422(2) | Mn | O5 | 2.347(2) |
| Cd16 | O15 | 2.228(2) | Mn | O7 | 2.062(2) |
| Cd16 | O4 | 2.3910(19) | Mn | O8 | 2.331(2) |

| (*R*)-1-Zn | | | (*R*)-1-Co | | |
| --- | --- | --- | --- | --- | --- |
| Atom | Atom | Length/Å | Atom | Atom | Length/Å |
| Zn1 | O7^1^ | 1.941(3) | Co | O2^1^ | 1.948(14) |
| Zn1 | O1^2^ | 1.984(3) | Co | O5 | 1.980(8) |
| Zn1 | O4 | 1.955(3) | Co | O6^2^ | 1.984(11) |
| Zn1 | O5 | 1.983(11) | Co | O8 | 1.919(17) |
| (*R*)-1-Cd | | | (*R*)-1-Mn | | |
| Atom | Atom | Length/Å | Atom | Atom | Length/Å |
| Cd | O1^1^ | 2.450(3) | Mn | O3^1^ | 2.3473(17) |
| Cd | O2^1^ | 2.421(2) | Mn | O4^2^ | 2.3147(15) |
| Cd | O3^2^ | 2.389(2) | Mn | O5 | 2.0696(16) |
| Cd | O4 | 2.192(2) | Mn | O6^1^ | 2.3303(17) |
| Cd | O5^2^ | 2.407(2) | Mn | O8 | 2.1290(15) |
| Cd | O8 | 2.226(2) | Mn | O9^2^ | 2.2842(15) |

## 4.2 Structure of (*S*)-1-Zn


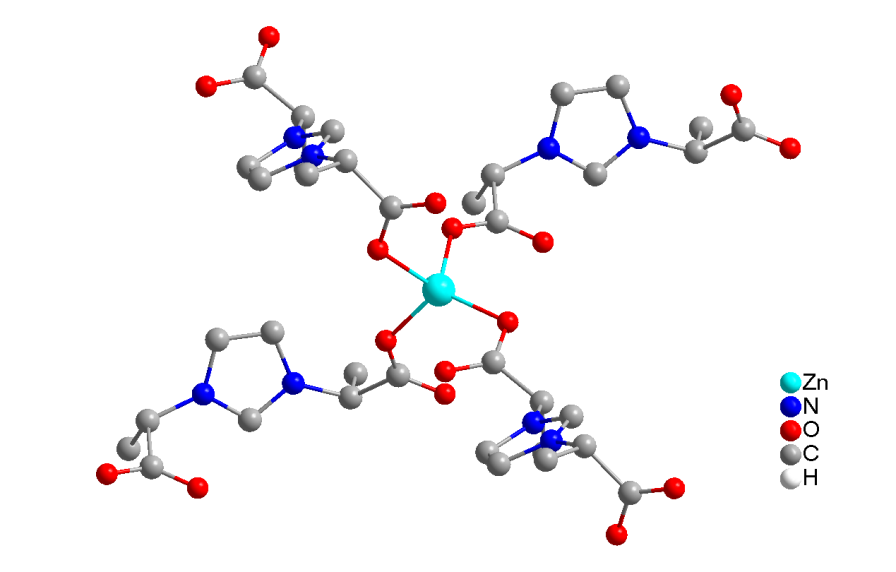


**Figure S2.** Structural unit of (*S*)-1-Zn


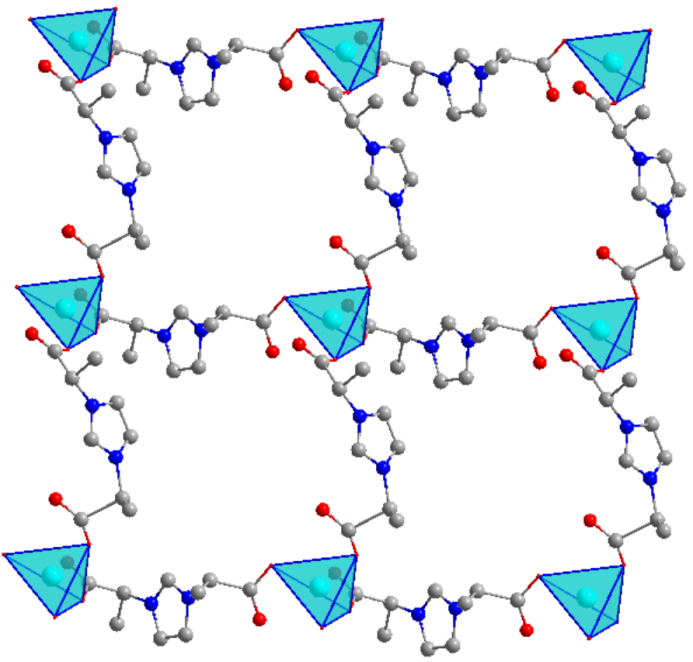


**Figure S3.** Layer structure of (*S*)-1-Zn


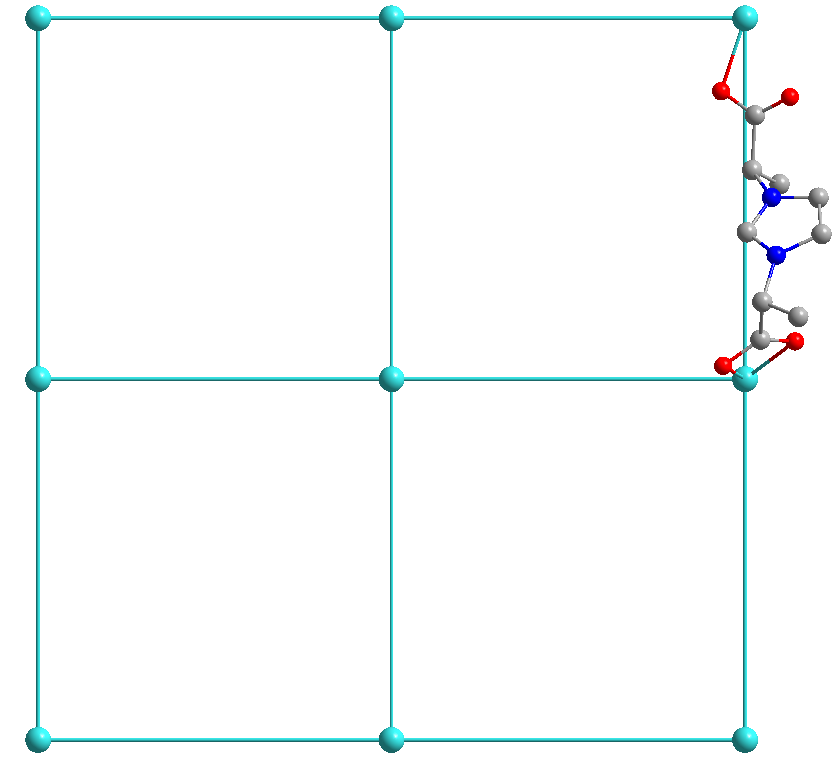


**Figure S4.** The simplified topology of (*S*)-1-Zn.

## 4.2 Circular dichroism (CD) spectra.


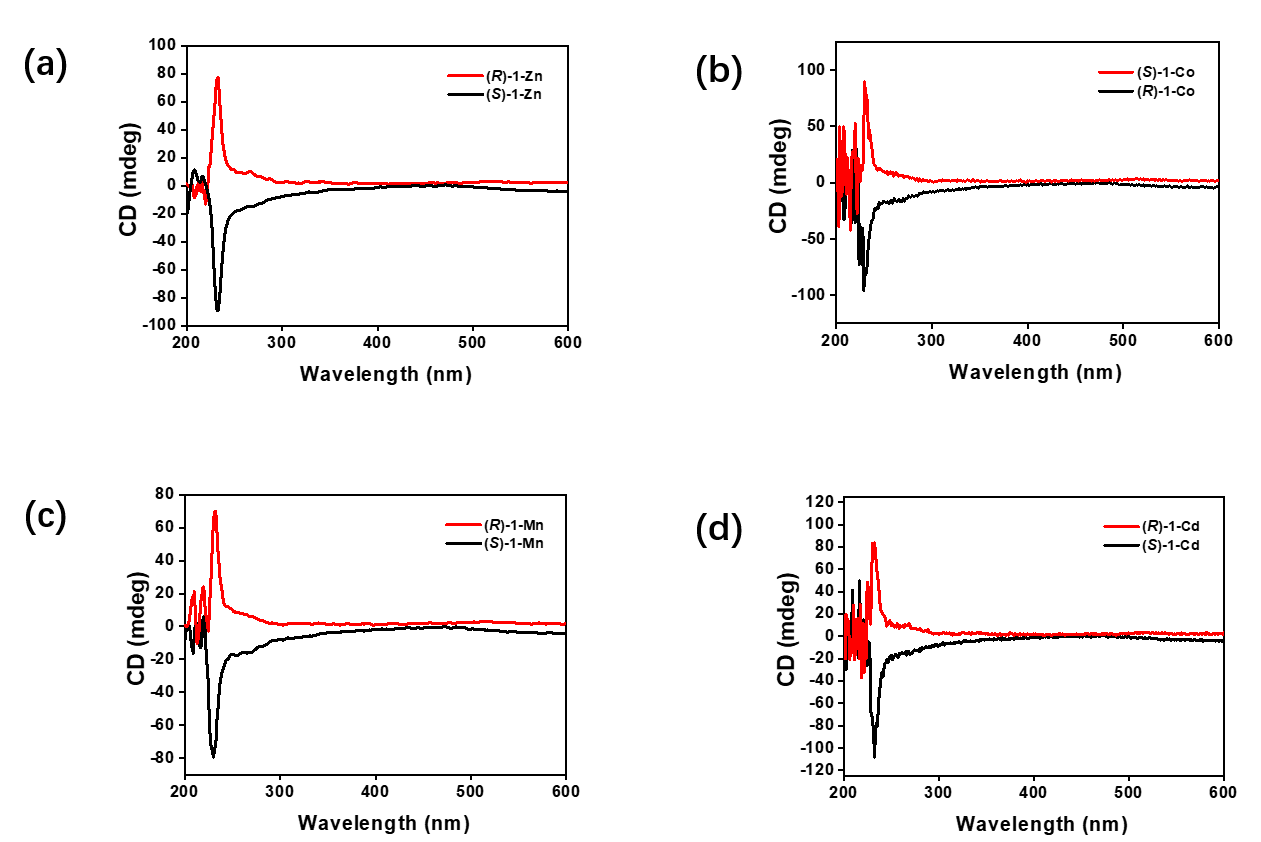


**Figure S5.** (a) CD curve of (*R*)/(*S*)-1-Zn. (b) CD curve of (*R*)/(*S*)-1-Co. (c) CD curve of (*R*)/(*S*)-1-Mn. (d) CD curve of (*R*)/(*S*)-1-Cd.

## 4.3 PXRD spectra.

**Figure S6.** PXRD patterns of (*S*)-1-Zn after immersed in organic solvents.

**Figure S7.** PXRD patterns of (*S*)-1-Zn after immersed in organic solvents for 3 months.


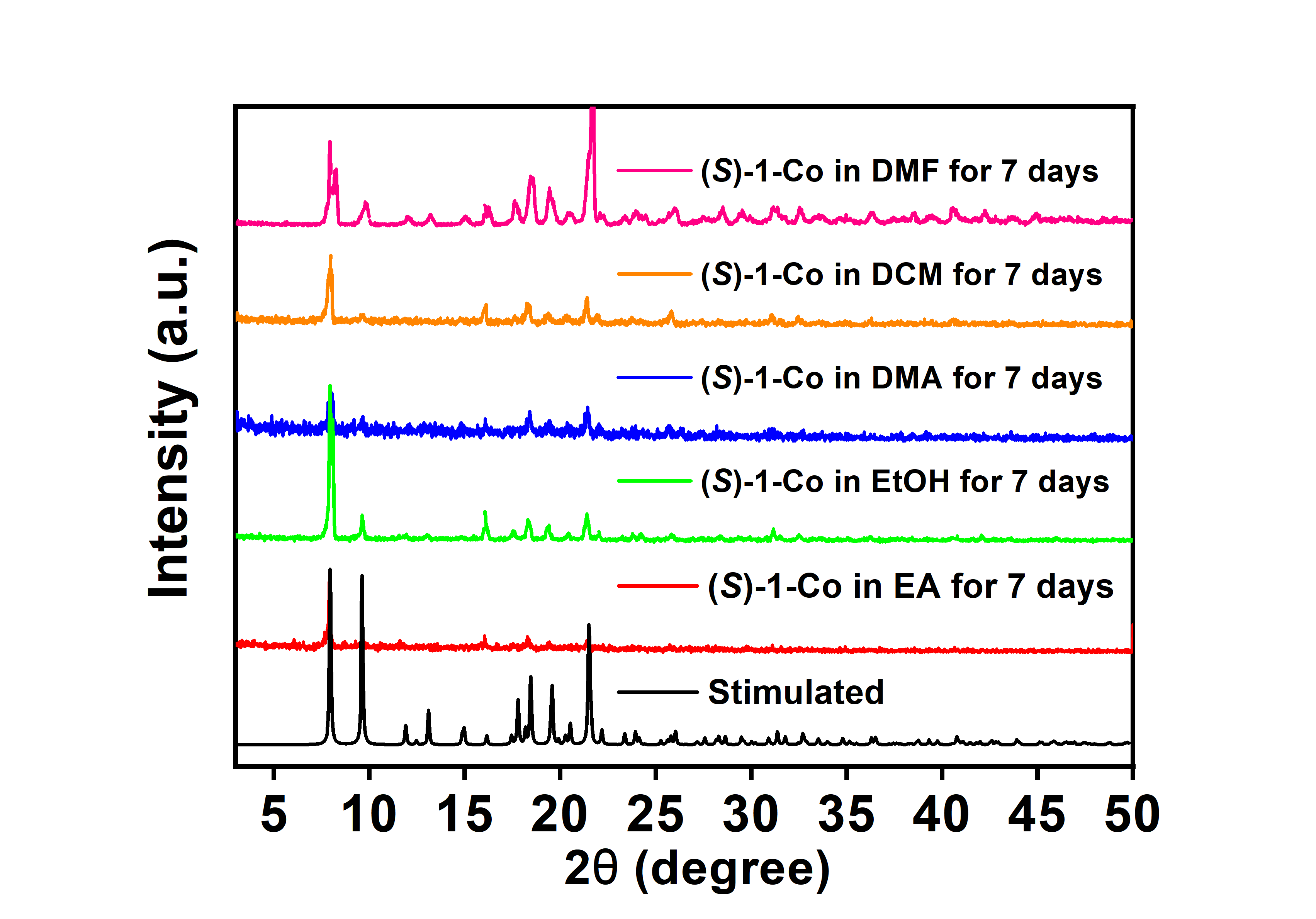


**Figure S8.** PXRD patterns of (*S*)-1-Co after immersed in organic solvents.


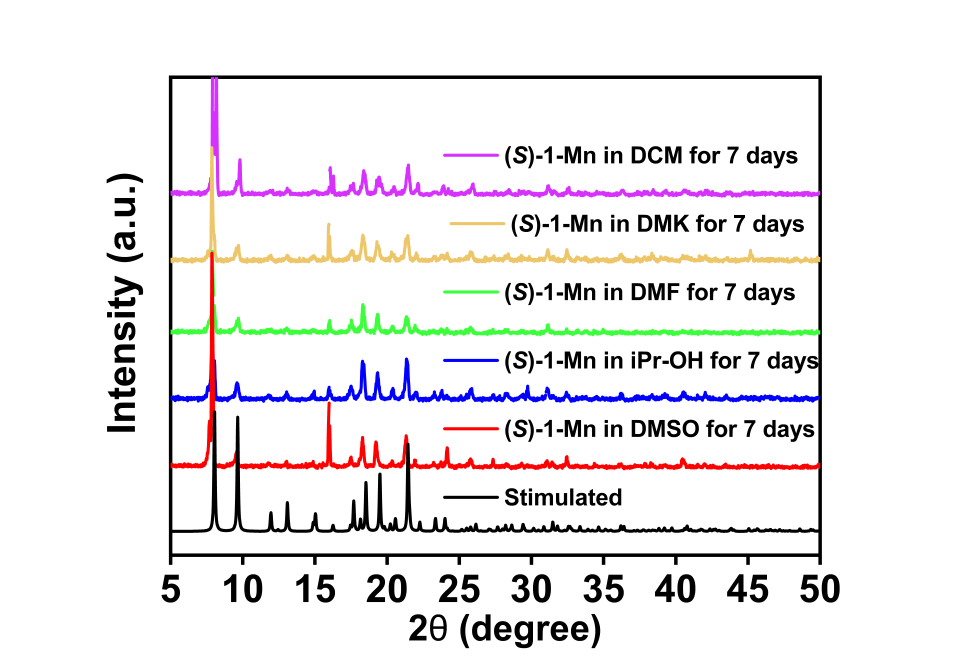


**Figure S9.** PXRD patterns of (*S*)-1-Mn after immersed in organic solvents.


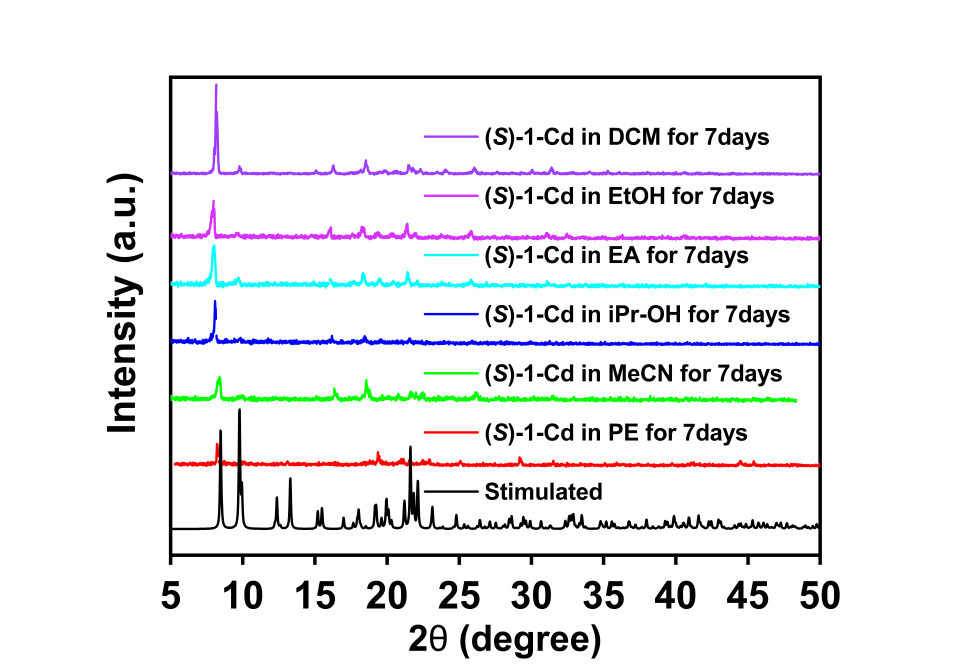


**Figure S10.** PXRD patterns of (*S*)-1-Cd after immersed in organic solvents.

## 4.4 TG spectra.


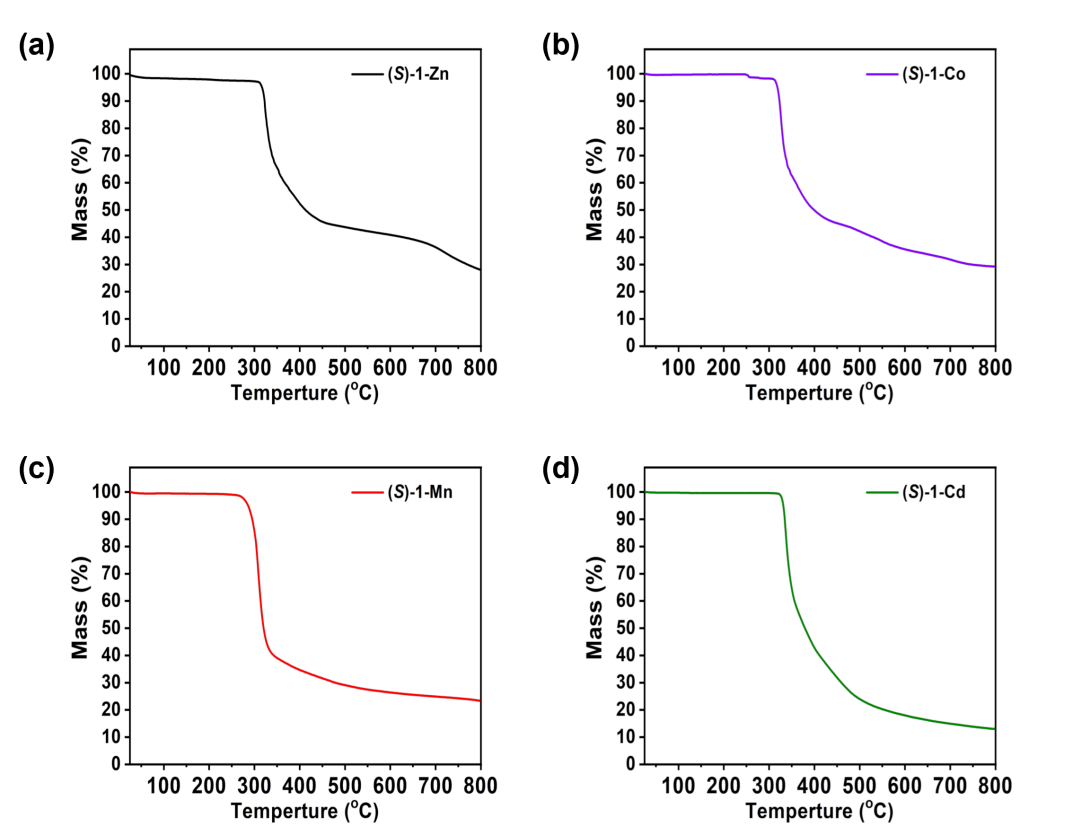


**Figure S11.** (a) The TG spectra of (*S*)-1-Zn. (b)The TG spectra of (*S*)-1-Co. (c) The TG spectra of (*S*)-1-Mn. (d) The TG spectra of (*S*)-1-Cd.

## 4.5 FTIR spectra.


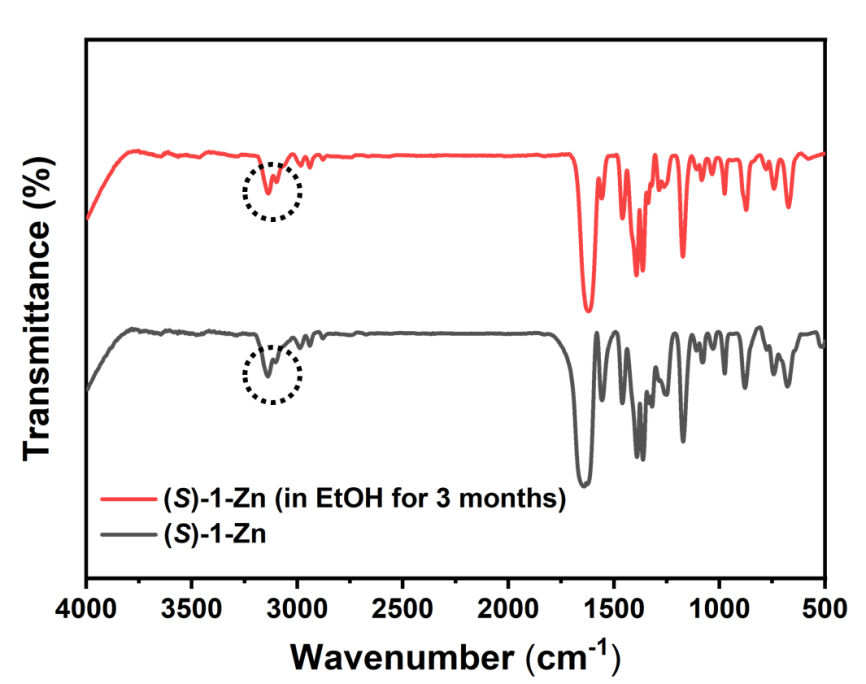


**Figure S12.** Fourier transform infrared spectroscopy of (*S*)-1-Zn.

# 5. Exfoliation of (*S*)-1-Zn

**5.1 General operation procedure**:

The newly synthesized bulk (*S*)-1-Zn (200 mg) was dispersed in a 500 mL screw-cap bottle, followed by the addition of EtOH (300 mL) and TBAB (5 mg). The resulting mixture was subjected to sonication at room temperature for 2 hours, yielding a homogenous milk-white dispersion (Notice: The temperature of process should be kept stable). Subsequently, the dispersion was allowed to sediment for 24 hours before being centrifuged at 9000 rpm for 5 minutes to efficiently remove any remaining non-exfoliated powders. To further purify the product, the sedimented dispersion was washed with EtOH (3 mL), followed by repeated centrifugation steps three times. Finally, the upper colloidal layer, containing the MON, was collected, achieving an isolated yield of approximately 8 wt%.

## 5.2 SEM spectra.


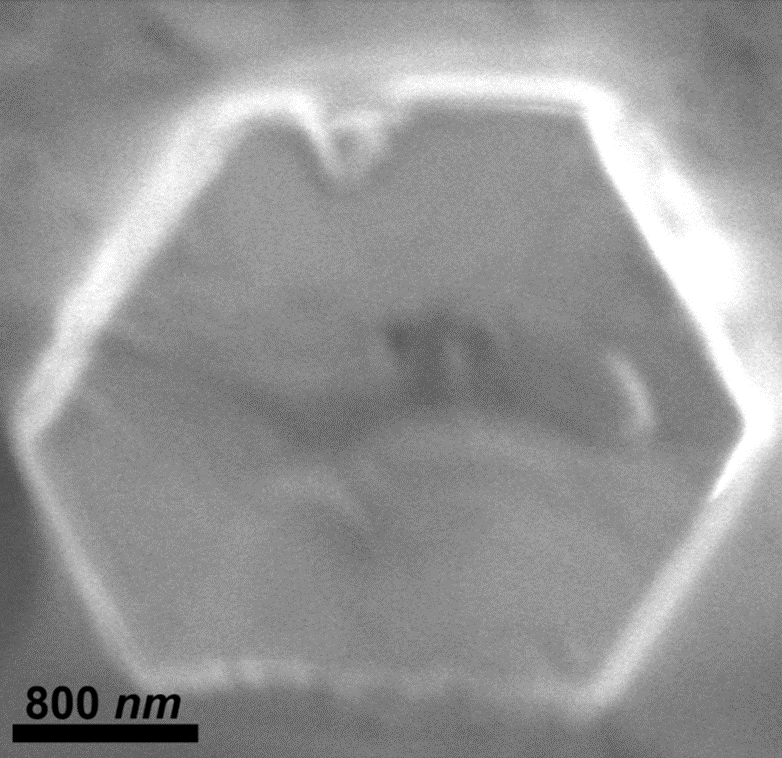


**Figure S13.** SEM spectra of (*S*)-1-Zn-MON.

# 6. Asymmetric Catalysis

## 6.1 General procedure for the experiments.

Before catalysis, the MOFs or MONs underwent an exchange process with EtOH for two days, followed by activation at 60°C for 4 hours to eliminate guest solvents. To a flame-dried Schlenk tube, the activated catalyst (2.0 mol%, relative to the substrate), *p*-anisaldehyde (200 μL, 2 mmol), proton sponge (5.0 mol%, relative to the substrate), and EtOH (2 mL) were added. The resulting mixture was vigorously stirred at 60 °C for 48 hours under an inert N_2_ atmosphere. After completion, the mixture was concentrated under reduced pressure and subjected to column chromatography on silica gel using a DCM/petroleum ether mixture (1:1, v/v) as the eluent to isolate the desired products. The enantiomeric excess (ee) value of the products was determined by HPLC analysis.

## 6.2 More reaction conditions screenings.

**Table S3**. Condition optimizations for asymmetric benzoin condensation. ^[a]^

| Entry | solvent | Base | Yield (%) | ee (%) |
| --- | --- | --- | --- | --- |
| 1 | EtOH | K_2_CO_3_ | trace | - |
| 2 | EtOH | KOH | N.D. | - |
| 3 | EtOH | Et_3_N | N.D. | - |
| 4 | EtOH | *^i^*Pr_2_NEt | trace | - |
| 5 | EtOH | DBU | 28 | 11 |
| 6 | EtOH | Proton Sponge | 47 | 45 |
| 7 | DCM | Proton Sponge | trace | - |
| 8 | THF | Proton Sponge | N.D. | - |
| 9 | DMF | Proton Sponge | N.D. | - |
| 10 | MeOH | Proton Sponge | 5 | 4 |
| 11 | DCE | Proton Sponge | trace | - |

1. Reaction conditions: *p*-Anisaldehyde (2 mmol, 1.0 equiv), (*S*)-1-Zn (0.04 mmol, 2 mol%), base (0.1 mmol, 5mol%), solvent (2 mL), N_2_ atmosphere, 60 ^o^C, 48 h. Isolated yields was reported. Enantiomeric ratios of **1a** were determined by HPLC analysis on a chiral stationary phase.

## 6.3 Recycle of the nanosheets of (*S*)-1-Zn.

**General operation procedure**:

To a flame-dried Schlenk tube was added activated nanosheets of (*S*)-1-Zn (2.0 mol%, based on the product), *p*-Anisaldehyde (200 μL, 2 mmol), proton sponge (5.0 mol%, based on the substrate), and EtOH (2 mL). The mixture was stirred at 60 ^o^C for 48 h under N_2_ atmosphere. After that, NH_4_PF_6_ solution (5% in EtOH) was added, and the mixture sonicated at room temperature for 1 h and centrifuged at 9000 rpm for 2 min. The supernatant was collected and concentrated under vacuum, and then the precipitate was washed with CHCl_3_ until no product in the was detected in the eluent. The rest of precipitate was reactivated at 60 ^o^C for 6 h, and then reused for the next runs directly.


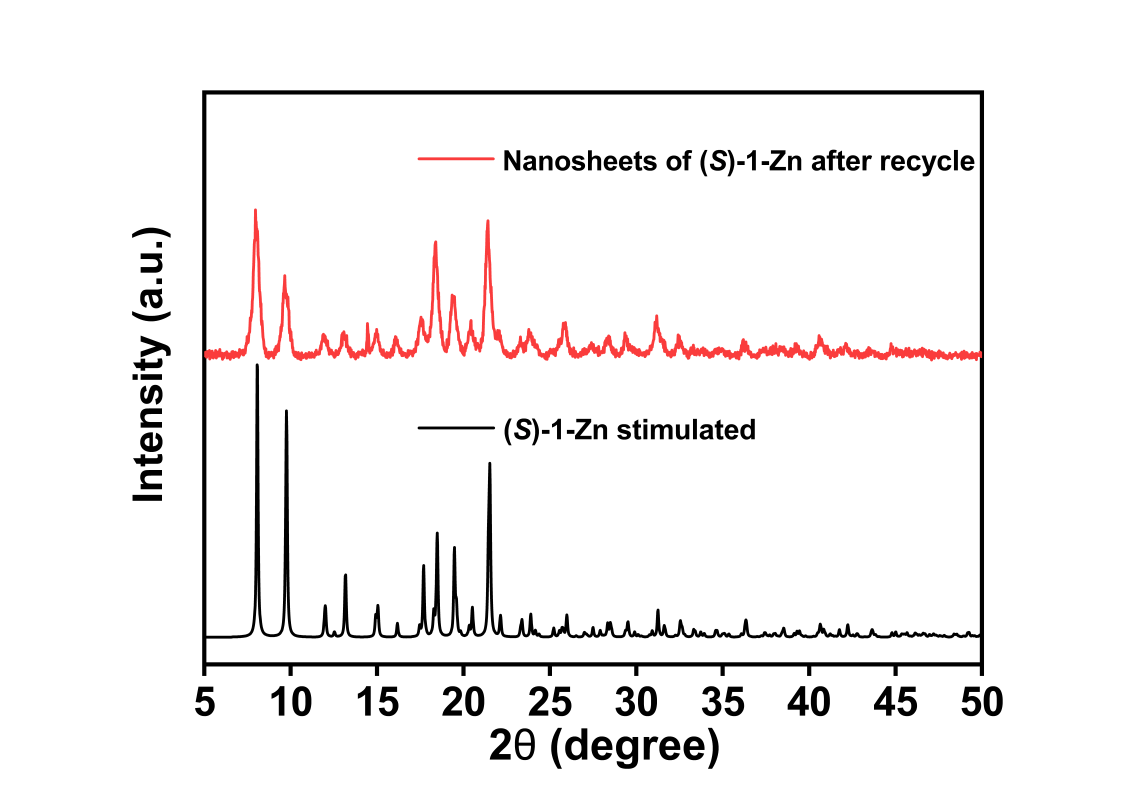


**Figure S14.** PXRD spectra of Nanosheets of (*S*)-1-Zn and bulky of (*S*)-1-Zn after recycle.

## 6.4 NMR and HPLC spectra of the products.

**2-hydroxy-1,2-bis(4-methoxyphenyl)ethan-1-one：** ^1^H NMR (400 MHz, CDCl_3_) δ 7.90 (dt, *J* = 9.1, 2.4 Hz, 2H), 7.25 (dd, *J* = 8.8, 1.9 Hz, 2H), 6.85 (dq, *J* = 7.6, 2.4, 2.0 Hz, 4H), 5.85 (dd, *J* = 6.2, 1.8 Hz, 1H), 4.59 (dd, *J* = 6.0, 1.9 Hz, 1H), 3.81 (d, *J* = 1.6 Hz, 3H), 3.75 (d, *J* = 1.7 Hz, 3H). ^13^C NMR (101 MHz, CDCl_3_) δ 197.44, 164.10, 159.75, 131.97, 131.70, 129.14, 126.40, 114.62, 114.04, 75.37, 55.61, 55.37. Analytical method for HPLC: Chiralcel OD-H column (hexane / *i*-PrOH = 80 / 20, 1.0 mL/min, 254 nm), t_1_ = 13.687 min, t_2_ = 14.912 min.

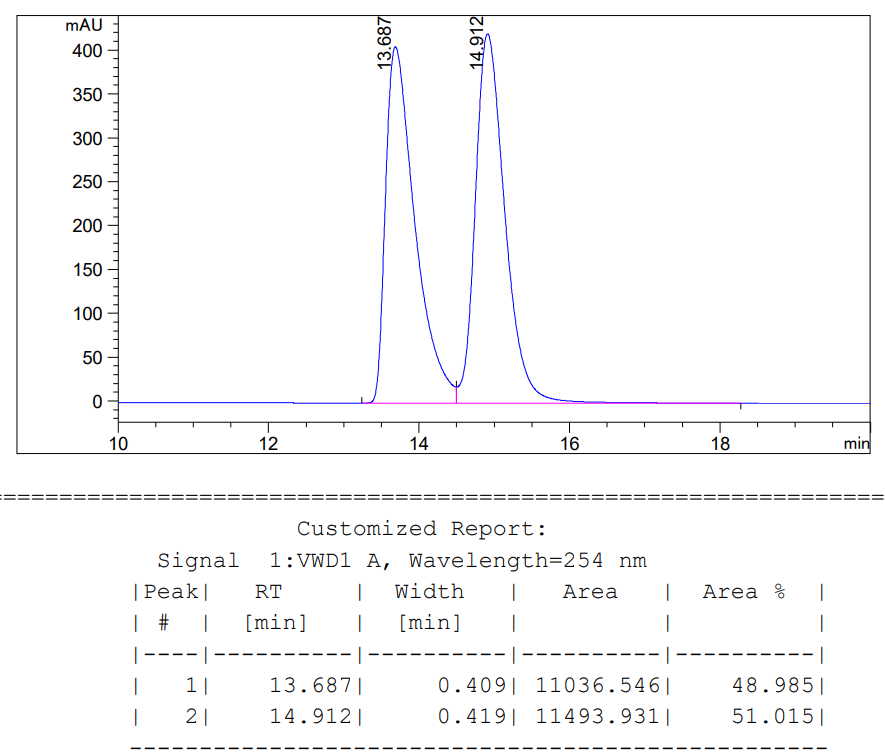

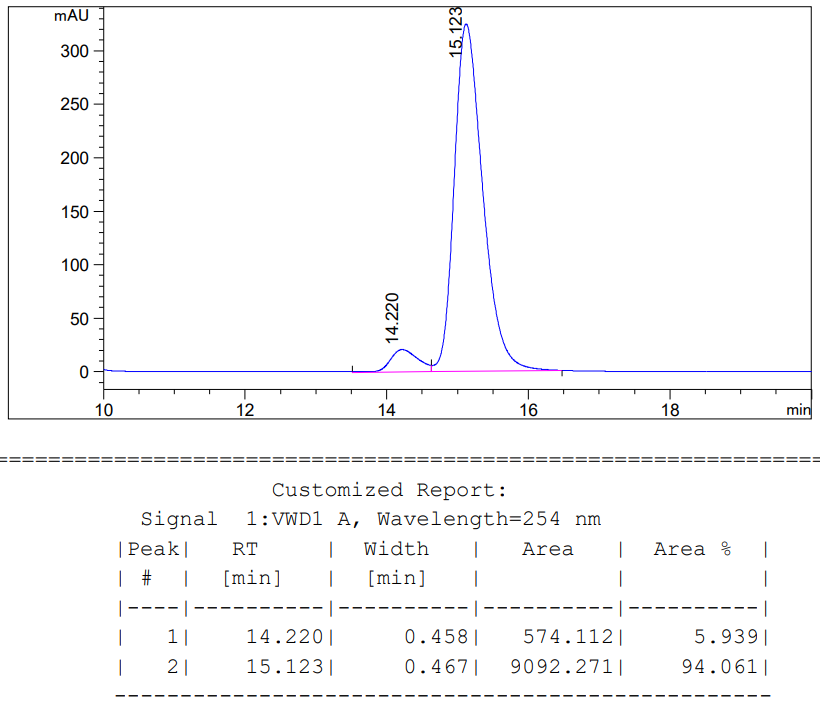


**Figure S15.** NMR and HPLC spectra of **1a**.

**2-hydroxy-1,2-diphenylethan-1-one:** ^1^H NMR (400 MHz, Chloroform-*d*) δ 7.98 – 7.82 (m, 2H), 7.60 – 7.46 (m, 1H), 7.45 – 7.17 (m, 8H), 5.95 (d, *J* = 5.8 Hz, 1H), 4.73 – 4.47 (m, 1H).^13^C NMR (101 MHz, Chloroform-*d*) δ 199.05, 139.10, 134.03, 133.56, 129.26, 129.24, 128.80, 128.69, 127.88, 76.31. Analytical method for HPLC: Chiralcel OD-H column (hexane / *i*-PrOH = 80 / 20, 1.0 mL/min, 254 nm), t_1_ = 8.057 min, t_2_ = 10.927 min.

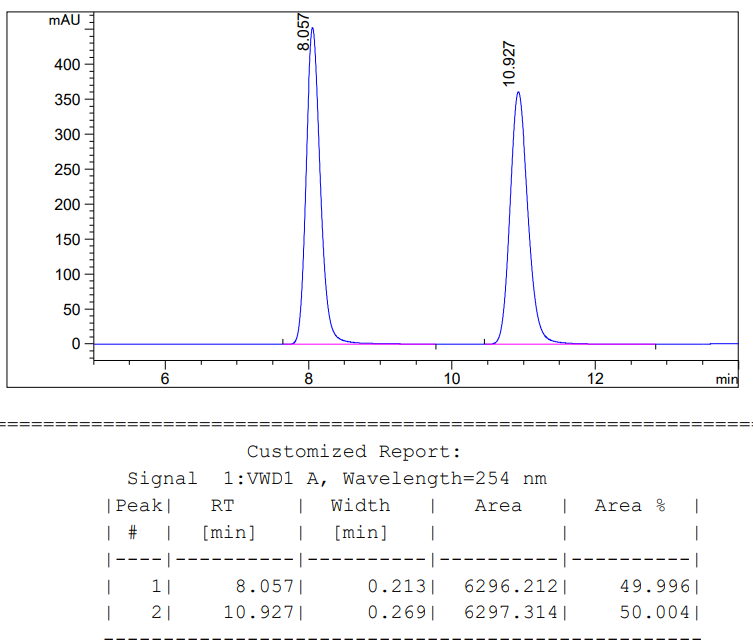

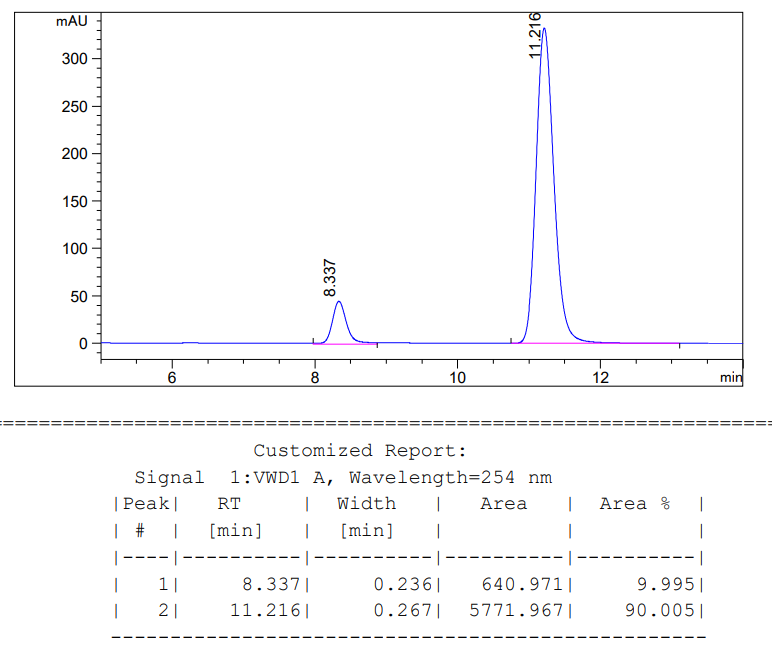


**Figure S16.** NMR and HPLC spectra of **1b**.

**2-hydroxy-1,2-di-p-tolylethan-1-one:** ^1^H NMR (400 MHz, CDCl_3_) δ 7.84 – 7.78 (m, 2H), 7.24 – 7.14 (m, 4H), 7.11 (d, *J* = 7.7 Hz, 2H), 5.89 (s, 1H), 4.56 (s, 1H), 2.34 (s, 3H), 2.28 (s, 3H). ^13^C NMR (101 MHz, CDCl_3_) δ 198.69, 145.06, 138.48, 136.50, 131.05, 129.93, 129.51, 129.42, 127.79, 75.93, 21.87, 21.30. Analytical method for HPLC: Chiralcel OD-H column (hexane / *i*-PrOH = 80 / 20, 1.0 mL/min, 254 nm), t_1_ = 6.335 min, t_2_ = 7.659 min.

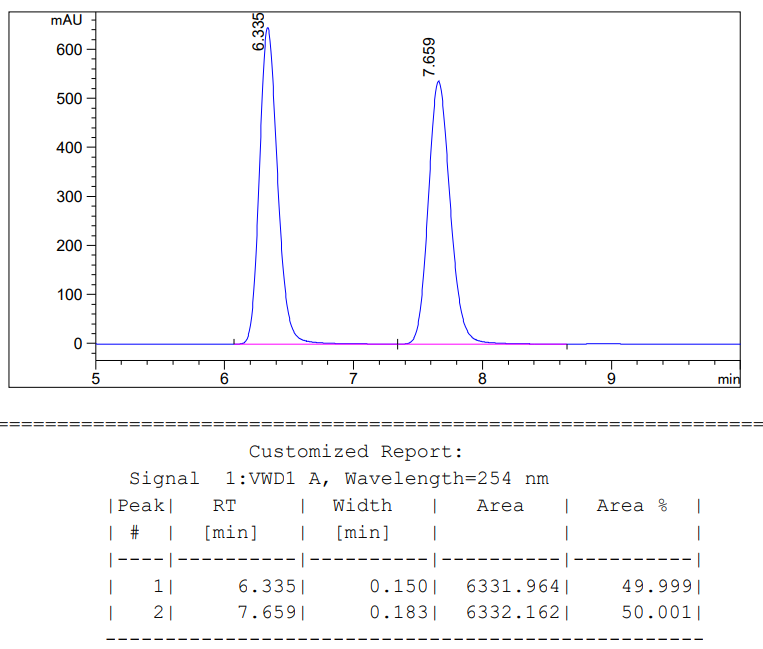

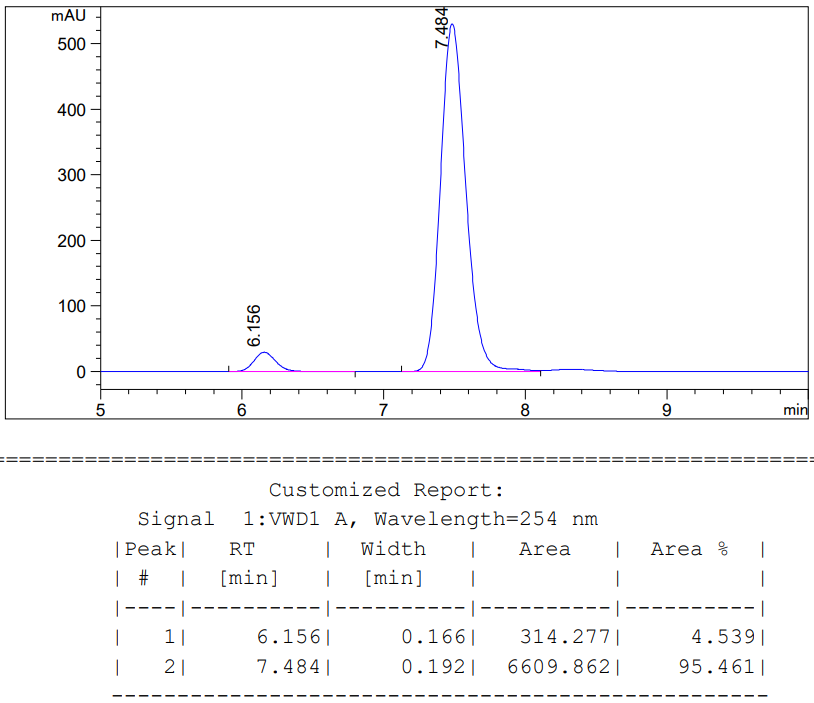


**Figure S17.** NMR and HPLC spectra of **1c**.

# 7. EPR test

**General procedure**：Under an inert N_2_ atmosphere, to a quartz sample tube, (*S*)-1-Zn MONs (1 mg), *p*-anisaldehyde (40 μL) and EtOH (0.4 mL) were added. Finally added proton sponge (1 mg) and sealed the sample tube. Subsequently, EPR experiments were performed at room temperature.

# 8. Reference

1. Caroline L. W, Fre´de´ric G, Julien P, Sylvain Rd, Pierre M, Alexandre A. Enantioselective copper catalysed 1,4-Conjugate Addition reactions using chiral N-Heterocyclic Carbenes. *Journal of Organometallic Chemistry*. **2005**, 690(24-25): 5672-5695.
2. Francisco L, Esther C, Ana O, Josep M, Further evidences about the role of bis(thiazolin-2-ylidene)s as the actual catalytic species in the generalised benzoin condensation. *Tetrahedron Letters*,**1996**, 37, 28, 5019 - 5022
3. Louise, B, Christopher A, R; Kirsten, Z; Stephen J, C. Highly Enantioselective Benzoin Condensation Reactions Involving a Bifunctional Protic Pentafluorophenyl-Substituted Triazolium Precatalyst. *J. Org. Chem*. **2009**, *74*, 9214–9217.
